# 2-Cys peroxiredoxin is required in successful blood-feeding, reproduction, and antioxidant response in the hard tick *Haemaphysalis longicornis*

**DOI:** 10.1186/s13071-016-1748-2

**Published:** 2016-08-19

**Authors:** Kodai Kusakisako, Remil Linggatong Galay, Rika Umemiya-Shirafuji, Emmanuel Pacia Hernandez, Hiroki Maeda, Melbourne Rio Talactac, Naotoshi Tsuji, Masami Mochizuki, Kozo Fujisaki, Tetsuya Tanaka

**Affiliations:** 1 Laboratory of Infectious Diseases, Joint Faculty of Veterinary Medicine, Kagoshima University, Korimoto, Kagoshima 890-0065 Japan; 2 Department of Pathological and Preventive Veterinary Science, United Graduate School of Veterinary Science, Yamaguchi University, Yoshida, Yamaguchi 753-8515 Japan; 3 Department of Veterinary Paraclinical Sciences, College of Veterinary Medicine, University of the Philippines Los Baños, Los Baños, Laguna 4031 Philippines; 4 National Research Center for Protozoan Diseases, Obihiro University of Agriculture and Veterinary Medicine, Inada-cho, Obihiro, Hokkaido 080-8555 Japan; 5 Department of Parasitology, Kitasato University School of Medicine, Minami, Sagamihara, Kanagawa 252-0374 Japan; 6 National Agricultural and Food Research Organization, Tsukuba, Ibaraki 305-0856 Japan

**Keywords:** Peroxiredoxin, *Haemaphysalis longicornis*, RNA interference, Hydrogen peroxide

## Abstract

**Background:**

Ticks are obligate hematophagous arthropods that feed on vertebrate blood that contains iron. Ticks also concentrate host blood with iron; this concentration of the blood leads to high levels of iron in ticks. The host-derived iron reacts with oxygen in the tick body and this may generate high levels of reactive oxygen species, including hydrogen peroxide (H_2_O_2_). High levels of H_2_O_2_ cause oxidative stress in organisms and therefore, antioxidant responses are necessary to regulate H_2_O_2_. Here, we focused on peroxiredoxin (Prx), an H_2_O_2_-scavenging enzyme in the hard tick *Haemaphysalis longicornis*.

**Methods:**

The mRNA and protein expression profiles of 2-Cys peroxiredoxin (HlPrx2) in *H. longicornis* were investigated in whole ticks and internal organs, and developmental stages, using real-time PCR and Western blot analysis during blood-feeding. The localization of HlPrx2 proteins in tick tissues was also observed by immunostaining. Moreover, knockdown experiments of *HlPrx2* were performed using RNA interference to evaluate its function in ticks.

**Results:**

Real-time PCR showed that *HlPrx2* gene expression in whole ticks and internal organs was significantly upregulated by blood-feeding. However, protein expression, except in the midgut, was constant throughout blood-feeding. Knockdown of the *HlPrx2* gene caused significant differences in the engorged body weight, egg weight and hatching rate for larvae as compared to the control group. Finally, detection of H_2_O_2_ after knockdown of *HlPrxs* in ticks showed that the concentration of H_2_O_2_ significantly increased before and after blood-feeding.

**Conclusion:**

Therefore, HlPrx2 can be considered important for successful blood-feeding and reproduction through the regulation of H_2_O_2_ concentrations in ticks before and after blood-feeding. This study contributes to the search for a candidate target for tick control and further understanding of the tick’s oxidative stress coping mechanism during blood-feeding.

**Electronic supplementary material:**

The online version of this article (doi:10.1186/s13071-016-1748-2) contains supplementary material, which is available to authorized users.

## Background

In high concentrations, hydrogen peroxide (H_2_O_2_) is known to be a harmful chemical compound to aerobic organisms due to its ability to seriously damage membrane lipids, nucleic acids, and proteins [1]. Almost all aerobic organisms have developed defense systems to scavenge H_2_O_2_. Catalases, peroxidases, and peroxiredoxins (Prxs) are scavengers of H_2_O_2_ [2]. Prxs are ubiquitous antioxidant enzymes investigated in various organisms [3]. Particularly, high levels of Prxs are produced in mammalian cells, including erythrocytes [4]. Erythrocytes are exposed to more oxidative stress than any other cell type, due to the abundance of heme iron and oxygen, which can generate H_2_O_2_ [5]. These indicate that Prxs may have important roles in peroxide detoxification in cells.

Prxs can be divided into two groups according to the presence of one or two highly conserved cysteines in organisms, 1-Cys or 2-Cys Prx [3]. 2-Cys Prxs are identified by two conserved cysteines, peroxidatic and resolving cysteines [6]. On the other hand, the 1-Cys Prxs have a conserved peroxidatic cysteine and do not contain a resolving cysteine [7]. Enzymes of the Prx family exhibit antioxidant activity that catalyzes the reduction of H_2_O_2_ into water (H_2_O), with thioredoxin as an immediate hydrogen donor or donor thiol, respectively [7].

In some endoparasites, such as *Plasmodium* and *Fasciola* parasites, Prxs have been characterized as antigens or secreted proteins, suggesting that endoparasite Prxs may participate in interactions between the parasites and their hosts [8, 9]. Therefore, to evaluate the efficacy of antigens for these endoparasites, basic biological and bio-histological analyses such as mRNA and protein expression profiles, and the localization of proteins in these parasites have been studied.

Ticks need blood meals to develop from one stage to the next and for reproduction. Blood-feeding and the digestion of blood provide nutrition and energy for molting, development, and the vitellogenesis of ticks [10]. Ticks feed on vertebrate blood that contains iron, such as heme, ferrous iron, and other pro-oxidants. Ticks also concentrate host blood with iron; this concentration of the blood leads to high levels of iron in ticks. Host-derived iron may react with oxygen in the tick body, and then high levels of reactive oxygen species (ROS), including H_2_O_2_, may be generated [11]. *Haemaphysalis longicornis* 1-Cys Prx (HlPrx) has been reported previously; however, there is still little knowledge about the biological functions of Prxs in ticks [12].

In the present study, we analyzed mRNA and protein expression profiles and the localization of proteins in tick tissues of *H. longicornis* 2-Cys Prx, HlPrx2, identified previously [13]. Moreover, *HlPrx* and/or *HlPrx2* gene silencing was performed to clarify their functions in ticks using RNA interference. Finally, we demonstrated that the double knockdown of *HlPrx* and *HlPrx2* led to increased oxidative stress in ticks.

## Methods

### Ticks and animals

The parthenogenetic Okayama strain of *H. longicornis* has been maintained by blood-feeding on the ears of Japanese white rabbits (KBT Oriental Co. Ltd, Saga, Japan) in the Laboratory of Infectious Diseases, Joint Faculty of Veterinary Medicine, Kagoshima University [14]. Rabbits were cared for in accordance with the guidelines approved by the Animal Care and Use Committee of Kagoshima University (Approval no. VM13007) and maintained under regulated conditions throughout the experiments.

### Total RNA extraction and cDNA synthesis

To extract total RNA, whole ticks were homogenized using an Automill (Tokken, Chiba, Japan), while dissected organs were disrupted using a pellet pestle motor (Sigma-Aldrich, St. Louis, MO, USA). The extracted RNA was purified using TRI Reagent^Ⓡ^ (Sigma-Aldrich), and then treated with an RQ1 RNase-Free DNase (Promega, Madison, WI, USA). cDNA synthesis was performed with ReverTra Ace-α-^Ⓡ^ (Toyobo, Osaka, Japan) following the manufacturer’s protocol using 1 μg of total RNA.

### Expression analysis of *HlPrx2* mRNA

The expression analysis of the *HlPrx2* mRNA was performed with real-time PCR using THUNDERBIRD^Ⓡ^ SYBR^Ⓡ^ qPCR Mix (Toyobo) with a 7300 real-time PCR system (Applied Biosystems, Foster City, CA, USA). Gene-specific primers were designed to target *HlPrx2* and the internal control genes, as shown in Table 1. Standard curves were made from four-fold serial dilutions of the cDNA of adult ticks fed for three days. The PCR cycle profile was as follows: initial denaturation at 95 °C for 10 min, 40 cycles of a denaturation step at 95 °C for 15 s, and an annealing/extension step at 60 °C for 60 s. The data was analyzed with 7300 system SDS software (Applied Biosystems). At the first step of real-time PCR, *actin*, *tubulin*, *P0*, and *L23* genes were evaluated for standardization and *L23* was selected as the tick reference in the current study.

**Table 1:** Gene-specific primers used in this study

Primer	Sequence (5'–3')
HlPrx2 RT-F	TATGCCTAAGCTGGCGAAGC
HlPrx2 RT-R	CAGGCGAGGTGAGAGAAGTG
HlPrx RT-F	ATGAGGTCCTCCGTGCTACT
HlPrx RT-R	TGCCACACCGTCATAAGCAT
HlPrx2 real time-F	GTGTGCCCTGCTAACTGGAA
HlPrx2 real time-R	ATGAGACACACGGGGCTTTG
HlPrx2 T7-F	*TAATACGACTCACTATAGG* GATCAAGCTGTCCGATTACAAGAAC
HlPrx2 T7-R	*TAATACGACTCACTATAGG* TTCCAGTTAGCAGGGCACACT
HlPrx2 RNAi-F	GATCAAGCTGTCCGATTACAAGAAC
HlPrx2 RNAi-R	TTCCAGTTAGCAGGGCACACT
HlPrx T7-F	*TAATACGACTCACTATAGG* CACCACGGTTGGATCAAGGA
HlPrx T7-R	*TAATACGACTCACTATAGG* TTTGCAGAGCCACCACTCAA
HlPrx RNAi-F	CACCACGGTTGGATCAAGGA
HlPrx RNAi-R	TTTGCAGAGCCACCACTCAA
Actin RT-F	CCAACAGGGAGAAGATGACG
Actin RT-R	ACAGGTCCTTACGGATGTCC
Actin real time-F	ATCCTGCGTCTCGACTTGG
Actin real time-R	GCCGTGGTGGTGAAAGAGTAG
Tubulin real time-F	TTCAGGGGCCGTATGAGTAT
Tubulin real time-R	TGTTGCAGACATCTTGAGGC
P0 real time-F	CTCCATTGTCAACGGTCTCA
P0 real time-R	TCAGCCTCCTTGAAGGTGAT
L23 real time-F	CACACTCGTGTTCATCGTCC
L23 real time-R	ATGAGTGTGTTCACGTTGGC

Italics denote T7 RNA polymerase promoter sequences

### Production of an antiserum against recombinant HlPrx2

To prepare mouse anti-HlPrx2 sera, 100 μg of recombinant HlPrx2 (rHlPrx2; [13]) was completely mixed with Freund’s complete adjuvant (Sigma-Aldrich) and intraperitoneally injected into ddY female mice (four weeks old, Kyudo, Saga, Japan). After two weeks, these mice were injected with 100 μg of rHlPrx2 with Freund’s incomplete adjuvant (Sigma-Aldrich) twice at a two-week interval to boost the generation of antibodies against rHlPrx2. Their blood was collected two weeks after the third immunization to obtain specific antisera to rHlPrx2.

### Protein extraction and Western blot analysis

Homogenized ticks were suspended in phosphate buffered saline (PBS) and ultrasonicated three times, two minutes each (Vibra-Cell^TM^; Sonics and Materials, Newtown, CT, USA) on ice and finally centrifuged at 500× *g*. The supernatant was resolved in a 12 % SDS-PAGE gel under reducing conditions. After sodium dodecyl sulfate-polyacrylamide gel electrophoresis (SDS-PAGE), the proteins were transferred onto a polyvinylidene difluoride membrane (Immobilon^Ⓡ^-P; Millipore, Danvers, MA, USA). The membrane was blocked overnight with 3 % skim milk in PBS (pH 7.4) (blocking solution); it was incubated with a 1:500 dilution of anti-rHlPrx2 mouse sera in blocking solution at 37 °C for 1 h. For loading control, tubulin was detected using antiserum against recombinant *H. longicornis* tubulin [15]. After washing five times in PBS containing 0.05 % Tween 20 (PBS-T), the membrane was incubated with a 1:50,000 dilution of horseradish peroxidase (HRP)-conjugated sheep anti-mouse IgG (Dako, Glostrup, Denmark) in blocking solution at 37 °C for 1 h. After washing five times in PBS-T, bands were detected using Amersham^TM^ ECL^TM^ Prime Western Blotting Detection Reagent (GE Healthcare, Buckinghamshire, UK) and viewed using FluorChem^Ⓡ^ FC2 software (Alpha Innotech, San Leandro, CA, USA). To accurately determine differences in the protein expression, band densitometry analysis was performed using Alpha View Software (Alpha Innotech). The band densitometry analysis results shown in this study represent the mean of three trials of Western blot analysis.

### Immunostaining

To confirm the localization of HlPrx2 in tick tissues, indirect immunofluorescent antibody tests (IFAT) were performed. Engorged ticks were dissected under a stereomicroscope (SZX10, Olympus, Tokyo, Japan) for collecting tick internal organs. Dissected organs were fixed in a 4 % paraformaldehyde phosphate buffer solution (pH 7.4) that included 0.1 % glutaraldehyde at 4 °C overnight. After washing with a sucrose series, organs were embedded in a Tissue-Tek^Ⓡ^ O.C.T Compound (Sakura Finetek, Torrance, CA, USA). Frozen sections from each internal organ were cut to a thickness of 10 μm using Kawamoto's film method (Leica Microsystems, Tokyo, Japan) and a cryostat (Leica CM 1850, Leica Microsystems, Wetzlar, Germany). The films were blocked with 5 % skim milk in PBS (pH 7.4) (blocking solution) at 37 °C for 1 h, and then incubated with 1:50 dilution in a blocking solution of anti-HlPrx2 mouse serum at 37 °C for 1 h. For the negative control, normal mouse serum was used. After washing three times in PBS, the slides were incubated at 37 °C for 1 h with Alexa Fluor^Ⓡ^ 594 goat anti-mouse IgG (Invitrogen, Carlsbad, CA, USA) with 1:1,000 dilution in the blocking solution. After removing the antibody by washing three times with PBS, the films were placed on a glass slide and mounted with DAPI (VECTASHIELD^Ⓡ^; Vector Laboratories, Burlingame, CA, USA), and then covered with a cover glass. The images were recorded using a confocal laser scanning microscope (LSM700, Carl Zeiss, Jena, Germany). Hemocytes were prepared using a glass slide instead of film as described previously by Galay et al. [16]. Briefly, hemolymph collected from ticks by amputating the legs was smeared directly on glass slides and air-dried. After drying, hemocyte smears were fixed with 4 % paraformaldehyde in PBS that included 0.1 % glutaraldehyde. Thereafter, the same method used for the internal organs’ IFAT was performed.

### RNA interference (RNAi)

Two separate PCR reactions of approximately 469 bp with a single T7 promoter were generated using the following primer sets: a T7-attached gene-specific forward primer (HlPrx2 T7-F) and gene-specific reverse primer (HlPrx2 RNAi-R) and a T7-attached gene-specific reverse primer (HlPrx2 T7-R) and gene-specific forward primer (HlPrx2 RNAi-F) (Table 1). After gel purification of PCR products using a GENECLEAN^Ⓡ^ II KIT (MP Biomedicals, Irvine, CA, USA), double-stranded RNA of *H. longicornis* 2-Cys peroxiredoxin (*dsHlPrx2*) was synthesized using the T7 RiboMAX™ Express RNAi System (Promega) with two separate single-promoter templates in accordance with the manufacturer’s protocol. Double-stranded RNA of *H. longicornis* 1-Cys peroxiredoxin (*dsHlPrx*) was also synthesized using *HlPrx* gene-specific primers (Table 1). The firefly *luciferase* (*Luc*) gene [17] was used for control (*dsLuc* group). One microgram of *dsHlPrx*, *dsHlPrx2*, or *dsDouble* (*dsHlPrx* and *dsHlPrx2* were mixed at 1 μg concentration each) was injected into 30 unfed adult female ticks in each experimental group and *dsLuc* group through the fourth coxae into the hemocoel. Injected ticks were observed for one day and subsequently transferred to rabbits with each group infesting separate ears. Three to four days after attachment, three ticks were manually detached to confirm gene silencing using RT-PCR. The remaining ticks were allowed to feed until engorgement, and the total number of engorged ticks, the engorged body weight, the oviposition, and the hatching rate were assessed.

### Detection of hydrogen peroxide (H_2_O_2_) in adult female ticks during blood-feeding

The H_2_O_2_ concentration in ticks was measured using the ferrous oxidation of xylenol orange assay [18]. Briefly, homogenized unfed and partially fed ticks were suspended in 200 μl of Milli-Q H_2_O, while homogenized engorged ticks were suspended in 900 μl of Milli-Q H_2_O. The samples were centrifuged at 500× *g*, and the supernatant was collected. The supernatant from the engorged ticks was further diluted 10 times in Milli-Q H_2_O. Ninety microliters of the supernatant from unfed and partially fed ticks or the diluted supernatant from engorged ticks was used for a sample solution as described later. The assay reagent consisted of 125 μM xylenol orange, 250 μM ammonium iron (II) sulfate, 100 mM sorbitol, and 25 mM sulfuric acid. One hundred microliters of the sample solutions was added to a 1-ml assay reagent. The mixture was vortexed immediately, left at room temperature for 30 min, and measured at 560 nm using a spectrophotometer (Ultrospec 2100 pro; GE Healthcare, Pittsburgh, PA, USA). Finally, the ratio of the H_2_O_2_ concentration (μM) to the corresponding tick’s body weight (mg) was calculated.

### Statistical analysis

All experiments were conducted in two or three separate trials. Data except for hatching rate were statistically analyzed using Welch’s *t*-test. Hatching rate analysis was done using the chi-square test. *P* < 0.05 and *P* < 0.01 were considered to be statistically significant *vs* control.

## Results

### Transcription profiles of *HlPrx2*

**Fig. 1:**
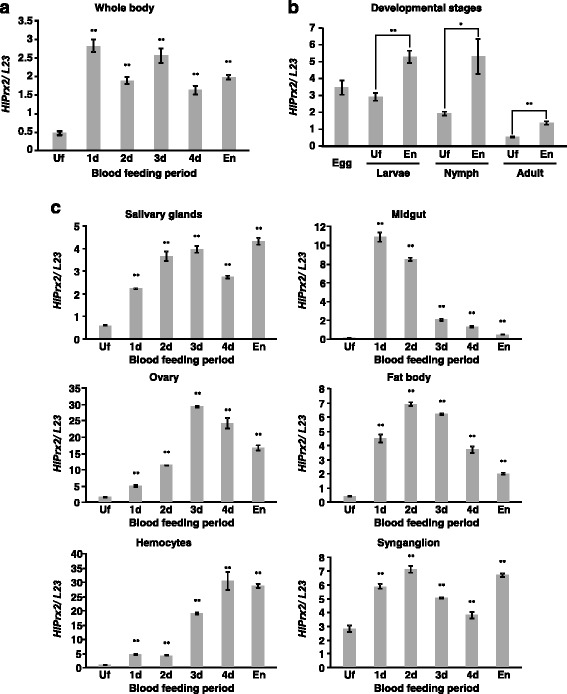
**a** Transcription profiles of *HlPrx2* in whole ticks during blood-feeding analyzed by real-time PCR (Uf, unfed females; 1d-4d, adults partially fed for 1–4 days). **b** Transcription profiles of *HlPrx2* in unfed and engorged tick developmental stages. **c** Transcription profiles of *HlPrx2* in the internal organs: salivary glands, midgut, ovary, fat body, hemocytes, synganglion). *L23* was used as the internal control. Data are presented as the mean ± standard deviation (SD). ^*^ *P* < 0.05; ^**^ *P* < 0.01, significant differences *vs dsLuc* by Welch’s *t*-test. *Abbreviations*: Uf, unfed ticks; En, engorged ticks

The mRNA levels of *HlPrx2* in whole female ticks and internal organs during blood-feeding and in different developmental stages (egg, larval, nymphal and adult stages) were investigated using real-time PCR. *HlPrx2* mRNA was upregulated in whole female ticks, developmental stages, and all internal organs (salivary glands, midgut, ovaries, fat bodies, synganglia and hemocytes) during blood-feeding (Fig. 1). In the whole body, mRNA was upregulated at day 1 and, in spite of higher expression levels as compared to those of unfed stage (Welch’s *t*-test: *t*_(2)_ = 23.43, *P* = 0.002), gradually decreased thereafter (Fig. 1a). Upregulation of the mRNA level was also observed in the developmental stages from unfed to engorgement, and the immature stages, including the egg, showed higher expression levels as compared to the adult stage (Welch’s *t*-test: Larvae, *t*_(2)_ = 9.77, *P* = 0.002; Nymph, *t*_(2)_ = 5.65, *P* = 0.030; Adult, *t*_(2)_ = 12.39, *P* = 0.006) (Fig. 1b). In the midgut, mRNA drastically increased at day 1 (Welch’s *t*-test: *t*_(2)_ = 36.31, *P* = 0.001) and decreased thereafter (Fig. 1c, Midgut). In the ovary, the expression level gradually increased until day 2, then drastically increased at day 3, and decreased thereafter (Fig. 1c, Ovary) (Welch’s *t*-test: day 2, *t*_(2)_ = 81.42, *P* < 0.001; day 3, *t*_(2)_ = 174.42, *P* < 0.001). In the hemocytes, the expression level increased at day 1 and remained almost the same at day 2, drastically increased from day 3 to day 4 (Welch’s *t*-test: day 1, *t*_(2)_ = 34.28, *P* = 0.001; day 2, *t*_(2)_ = 40.81, *P* = 0.001; day 3, *t*_(2)_ = 86.14, *P* < 0.001; day 4, *t*_(2)_ = 16.25, *P* = 0.004), and then slightly decreased at the engorged state (Fig. 1c, Hemocytes). The expression levels of *HlPrx2* gene in ovaries and hemocytes were higher than those of other internal organs. In other tissues, such as the salivary glands, fat bodies and synganglia, mRNA was upregulated from unfed to day 1 and remained at a high level until engorgement (Fig. 1c). These results indicate that the mRNA of *HlPrx2* gene was upregulated in ticks by blood-feeding. The high levels of mRNA expression in the ovaries and hemocytes suggest that *HlPrx2* gene may be related to the reproduction and immune response of ticks.

### Protein expression profiles of HlPrx2

**Fig. 2:**
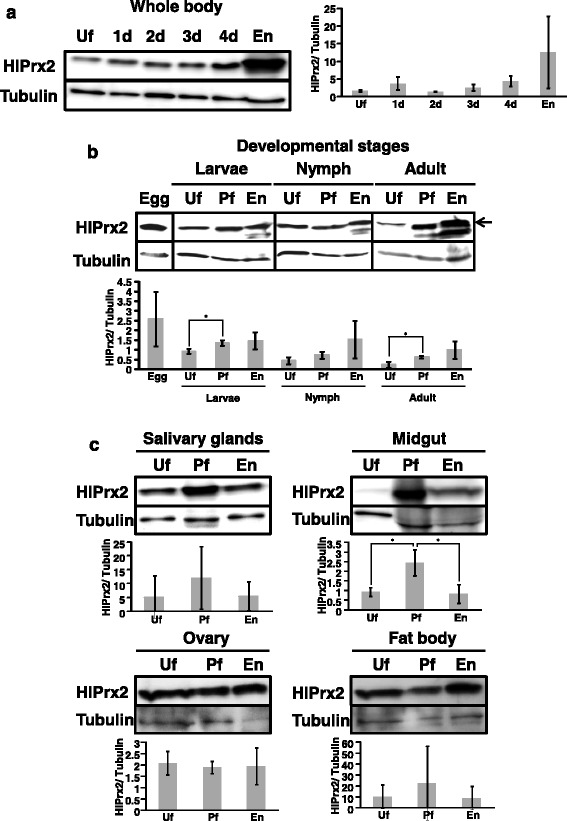
**a** Protein expression profiles of HlPrx2 in whole ticks during blood-feeding as analyzed by Western blot analysis. **b** Protein expression profiles of HlPrx2 in developmental stages. *Arrow* indicates native HlPrx2 protein as distinguished from the non-specific bands below. **c** Protein expression profiles of HlPrx2 in the internal organs (salivary glands, midgut, ovary, fat body, hemocytes, and synganglion). For loading control, tubulin was detected. The bars show the results of band densitometry analysis for HlPrx2. The relative expression was calculated based on tubulin. Data are presented as the mean ± standard deviation (SD). ^*^ *P* < 0.05, significant differences by Welch’s *t*-test. *Abbreviations*: Uf, unfed adults; Pf, partially fed adults at day 3; En, engorged adults

The protein expression levels of HlPrx2 in whole female ticks and internal organs during blood-feeding and in different developmental stages were investigated by Western blot analysis using HlPrx2-specific antisera. The predicted molecular mass of HlPrx2 protein is approximately 22 kDa, and the theoretical isoelectric point (pI) is 6.8; the signal peptide and glycosylation sites were not found with sequence analysis [13]. However, the calculated molecular mass in Western blot analysis was approximately 26 kDa. The mobility of native HlPrx2 protein in Western blot analysis decreased because the pI = 6.8 is slightly low. HlPrx2 expression was generally upregulated during blood-feeding in the whole body, the developmental stages, and the midgut (Fig. 2). In the whole body and the developmental stages, the HlPrx2 expression level was upregulated from unfed to engorgement (Fig. 2a, b). Notably, in the developmental stages, protein expression levels seemed to be almost the same, although immature stages, including the egg, showed higher mRNA expression levels as compared to those of the adult stage (Figs. 1b and 2b). In Fig. 2b, other bands under HlPrx2 band at the engorged state of all stages can be seen. These bands are considered to be non-specific bands derived from the blood of the host rabbit (see Additional file 1: Figure S1). These non-specific bands in the rabbit blood cross-reacted with HlPrx2 antisera; thus, these are considered to be a candidate cross-reacting protein related to 2-Cys peroxiredoxin (see Additional file 1: Figure S1 and Additional file 2). Moreover, in the knockdown ticks, the band of HlPrx2 protein was decreased as compared to the control group (see Additional file 1: Figure S2). Therefore, the anti-HlPrx2 mouse serum used in this study was considered as specifically working.

In the midgut, although the protein expression level was very low in the unfed stage, it significantly increased from unfed to partially fed states (Welch’s *t*-test: *t*_(2)_ = 3.66, *P* = 0.049) and significantly decreased in the engorged state (Welch’s *t*-test: *t*_(2)_ = 3.34, *P* = 0.034) (Fig. 2c, Midgut). In the salivary glands, ovaries, and fat bodies, the protein expression levels of HlPrx2 were constant during blood-feeding (Fig. 2c). These results indicate that the protein expression of HlPrx2 is strongly upregulated in the whole body, especially in the midgut, by blood-feeding; however, the expression levels of HlPrx2 protein in the other tissues, such as the salivary glands, ovaries and fat bodies, were constant during blood-feeding. The drastic increase of HlPrx2 protein expression in the midgut during blood-feeding suggests that HlPrx2 protein could be related to the antioxidant response in this tissue because ticks’ midgut may be exposed to high concentrations of ROS during blood-feeding.

### Localization of HlPrx2 in the salivary glands, midgut, ovaries, and hemocytes from engorged adult female ticks

**Fig. 3:**
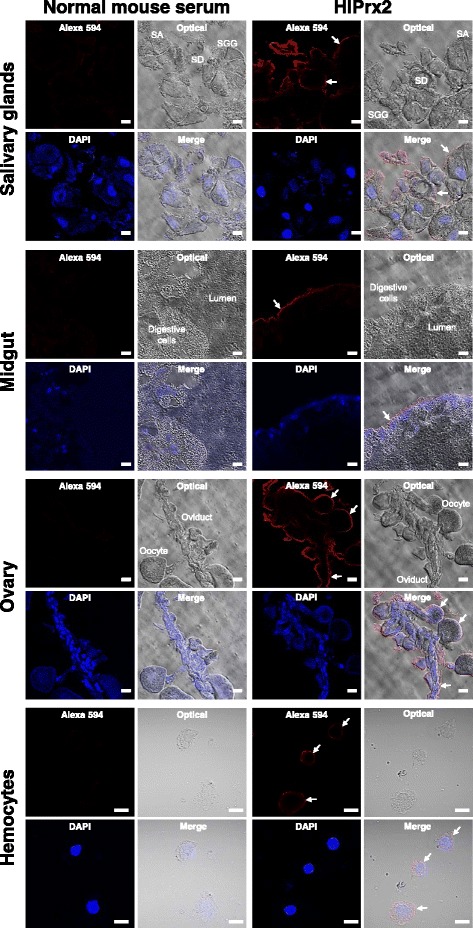
Localization of HlPrx2 proteins in the salivary glands, midgut, ovary, and hemocytes from engorged adult ticks using IFAT under a confocal laser scanning microscope. Anti-HlPrx2 mouse serum was used as a primary antibody. Anti-mouse IgG conjugated with Alexa Fluor 594 was used as a secondary antibody and nuclei were visualized using DAPI. Normal mouse serum was used for control. *Arrows* indicate the specific fluorescence. *Abbreviations*: SA, salivary gland acinar cells; SGG, salivary gland granular cells; SD, salivary duct. Scale-bars: 20 μm (salivary glands, midgut, and ovary) and 10 μm (hemocytes)

Western blot analysis showed the high expression of HlPrx2 protein in the whole body and internal organs. To determine localization in the cells of internal organs, IFAT was performed using some internal organs of engorged female ticks. In the salivary glands, positive fluorescence was detected in the cell membrane of the acinar cells (SA) and granular cells (SGG) and in the basal lamina of the salivary duct (SD) (Fig. 3, Salivary glands). In the midgut, positive fluorescence was detected in the basal lamina of the digestive cells (Fig. 3, Midgut). In the ovary, positive fluorescence was detected in the cell membrane of the oocytes and basal lamina of the oviduct (Fig. 3, Ovary), whereas in the hemocytes, positive fluorescence was detected in the cell membrane (Fig. 3, Hemocytes). These results demonstrate that the HlPrx2 protein was associated to the tissue membranes.

### Effects of *HlPrx* and/or *HlPrx2* gene silencing on the blood-feeding and reproduction of female ticks

**Fig. 4:**
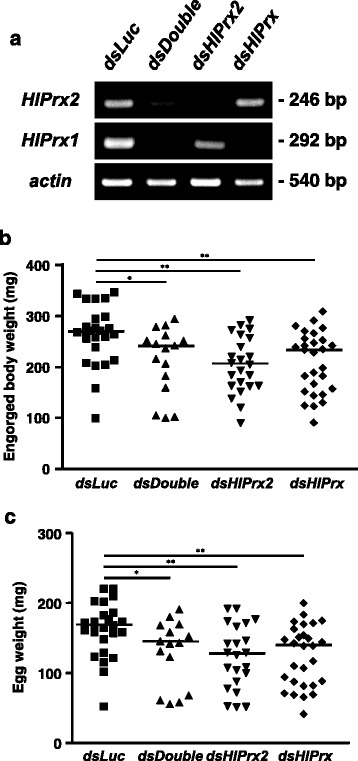
**a** Knockdown confirmation of *HlPrx* and/or *HlPrx2* genes in partially fed adult ticks. Each tick total RNA was extracted from 3 ticks pooled. The left column indicates the detection primer set; *actin* was used as a control. The right column indicates the size of the PCR products. **b** Column graph for engorged body weight in the knockdown experiment. **c** Column graph for egg weight after finishing oviposition by engorged adult ticks in the knockdown experiment. Horizontal lines indicate the median values. *Abbreviations*: *dsLuc*, double-stranded *Luciferase*-injected group; *dsHlPrx2*, double-stranded *HlPrx2*-injected group; *dsHlPrx*, double-stranded *HlPrx*-injected group; *dsDouble*, both double-stranded *HlPrx*- and *HlPrx2*-injected group. ^*^ *P* < 0.05; ^**^ *P* < 0.01, significant differences *vs dsLuc* by Welch’s *t*-test

To clarify the functions of the *HlPrx* and *HlPrx2* genes, gene silencing using the RNAi method was conducted. Gene silencing was confirmed by semi-quantitative RT-PCR and Western blot analysis (Fig. 4a). The knockdown of *HlPrx* and/or *HlPrx2* caused significant differences in the ticks’ engorged body weight, egg weight and hatching rate (Table 2). The ticks’ engorged body weight and egg weight significantly decreased (Fig. 4b and c). Notably, double knockdowns, wherein both *HlPrx* and *HlPrx2* were silenced, showed almost the same results as *HlPrx* silencing. *HlPrx2* silencing resulted in a greater decrease in engorged body weight and egg weight when compared to those of *dsDouble* and *dsHlPrx* silencing. However, the hatching rates of *dsHlPrx* and *dsHlPrx2* groups were similar (Table 2). These results suggest that the knockdown of *HlPrx* and/or *HlPrx2* genes significantly decreased engorged body weight, egg weight and hatching rate as compared to the *dsLuc* group.

**Table 2:** Effects of *HlPrx* and/or *HlPrx2* gene silencing in ticks

Knockdown groups	Infest No.	Drop No.	Engorged body weight (mg)	Egg weight (mg)	Ratio of egg weight/engorged body weight	Hatching rate (%)
*dsLuc*	30	25	263.7 ± 58.9	162.1 ± 38.9	0.61 ± 0.03	100
*dsDouble*	30	15	218.8 ± 66.2^a^	130.3 ± 46.7^d^	0.59 ± 0.08	87
*dsHlPrx2*	30	22	204.4 ± 56.3^b^	116.7 ± 45.3^e^	0.55 ± 0.09^g^	77^i^
*dsHlPrx*	30	28	210.0 ± 59.8^c^	124.8 ± 43.4^f^	0.58 ± 0.06^h^	78^j^

^a^Significant difference as compared with the *dsLuc* group by Welch’s *t*-test: *t*_(27)_ = 3.16, *P* = 0.020

^b^Significant difference as compared with the *dsLuc* group by Welch’s *t*-test: *t*_(45)_ = 3.53, *P* = 0.001

^c^Significant difference as compared with the *dsLuc* group by Welch’s *t*-test: *t*_(50)_ = 3.24, *P* = 0.002

^d^Significant difference as compared with the *dsLuc* group by Welch’s *t*-test: *t*_(26)_ = 2.22, *P* = 0.036

^e^Significant difference as compared with the *dsLuc* group by Welch’s *t*-test: *t*_(42)_ = 3.67, *P* = 0.001

^f^Significant difference as compared with the *dsLuc* group by Welch’s *t*-test: *t*_(51)_ = 3.30, *P* = 0.002

^g^Significant difference as compared with the *dsLuc* group by Welch’s *t*-test: *t*_(25)_ = 2.85, *P* = 0.009

^h^Significant difference as compared with the *dsLuc* group by Welch’s *t*-test: *t*_(41)_ = 2.46, *P* = 0.018

^i^Significant difference as compared with the *dsLuc* group by Chi-square test: χ^2^ = 6.36, *df* = 1, *P* = 0.012

^j^Significant difference as compared with the *dsLuc* group by Chi-square test: χ^2^ = 6.04, *df* = 1, *P* = 0.014

### Double knockdown of *HlPrx* genes increased the concentration of H_2_O_2_ before and after blood-feeding

**Fig. 5:**
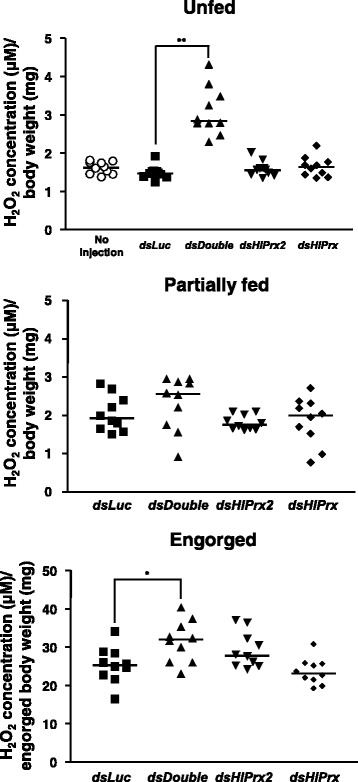
Concentrations of hydrogen peroxide (H_2_O_2_) from *HlPrx* and/or *HlPrx2* knockdown ticks during blood-feeding. Data are presented as the ratio of H_2_O_2_ concentration (μM) to tick body weight or engorged body weight (mg). Horizontal bars indicate the median value. *Abbreviations*: *dsLuc*, double-stranded *Luciferase*-injected group; *dsHlPrx2*, double-stranded *HlPrx2*-injected group; *dsHlPrx*, double-stranded *HlPrx*-injected group; *dsDouble*, both double-stranded *HlPrx*- and *HlPrx2*-injected group. ^*^ *P* < 0.05; ^**^ *P* < 0.01, significant differences *vs dsLuc* by Welch’s *t*-test

To elucidate the observed effects of *HlPrx* and/or *HlPrx2* gene silencing during blood-feeding, H_2_O_2_ concentrations were measured in female ticks. Gene silencing was also confirmed by semi-quantitative RT-PCR (data not shown). In the unfed and engorged states, the *dsDouble* group showed significantly higher concentrations of H_2_O_2_ as compared to the *dsLuc* group (Welch’s *t*-test: Unfed, *t*_(10)_ = 7.77, *P* < 0.001; Engorged, *t*_(17)_ = 2.72, *P* = 0.014) (Fig. 5, Unfed and Engorged). *HlPrx* or *HlPrx2* gene-silenced groups only showed slightly higher concentrations of H_2_O_2_ as compared to the *dsLuc*-injected group in the unfed state (Fig. 5, Unfed). On the other hand, in the engorged state, the *HlPrx2* gene-silenced group showed a slightly higher concentration of H_2_O_2_, whereas the *HlPrx* gene-silenced group showed a slightly lower concentration of H_2_O_2_ as compared to the *dsLuc*-injected group (Fig. 5, Engorged). These results demonstrate that the knockdown of *HlPrx* and *HlPrx2* genes leads to a high concentration of H_2_O_2_ in ticks before and after blood-feeding.

## Discussion

To protect against the toxicity of H_2_O_2_, aerobic organisms have evolved antioxidant enzymes, such as catalases, peroxidases, and Prxs [2]. Moreover, ticks lack heme synthesis and catabolism pathways because they are unable to prepare σ-aminolevulinic acid, a heme precursor, even at genomic levels [3, 19]. Therefore, they rely on heme from their host and store it in hemosomes of the midgut without digestion [4]. These facts suggest that ticks might face difficulties in producing proteins that contain heme, such as catalase and peroxidase, which are both H_2_O_2_-scavenging enzymes [20]. Moreover, ticks must acquire nutrients from the host blood meal and metabolize these nutrients via catabolism and anabolism [21]. *Plasmodium* parasites also take in nourishment from host blood and are likely to utilize members of the Prx family as the principal enzymes for reducing peroxides, including H_2_O_2_, because they lack catalase and peroxidase [22]. Therefore, Prxs might be similarly essential to the regulation of the H_2_O_2_ concentration for ticks.

In this study, we found that *HlPrx2* mRNA expression was upregulated by blood-feeding (Fig. 1). On the other hand, HlPrx2 protein expression was almost stable during blood-feeding, except in the midgut (Fig. 2). In the whole body, although mRNA expression was upregulated by blood-feeding when compared to the unfed state (Fig. 1a, b), protein expression seemed to be constant in all states of blood-feeding except for the engorged state, where it showed an increased expression level (Fig. 2a, b). *Fasciola* parasites secrete Prxs into their hosts to regulate their environment for survival in the host body [9]. Our results suggest that ticks may also secrete HlPrx2 protein into hosts as *Fasciola* parasites do, and the inconsistency of protein expression in comparison with mRNA expression may be due to the release of HlPrx2 proteins. Protein expression in the whole body increased according to the state of engorgement (Fig. 2c). This drastic change seems to be related to body size, because tick body weight notably increases from day 4 to engorgement, and the increase in body weight is about 100-fold compared to unfed ticks [23]. It may be also in response to the very large amounts of blood ingested during the rapid engorgement stage, which may expose ticks to higher levels of ROS. Although other developmental stages (larval and nymphal stages) also showed similar tendencies in HlPrx2 protein expression (Fig. 2b), *HlPrx2* mRNA expression in larval and nymphal stages was higher than in the adult stage (Fig. 1b). This result suggests that HlPrx2 protein might have an important role in the molting and survival of immature stages during blood-feeding and after engorgement.

In the internal organs, especially the midgut, HlPrx2 mRNA and protein expression was consistent (Figs. 1c and 2c). The mRNA and protein expression levels were negligible in the unfed midgut (Figs. 1c and 2c). Blood-feeding acts as a trigger, inducing the upregulation of HlPrx2 mRNA and protein expression. In IFAT examination of the midgut, HlPrx2-specific fluorescence was detected in the basal lamina (Fig. 3). There have been some reports that the multimer of 2-Cys Prxs are associated with membranes, such as red blood cells [24, 25]. Our results, along with those of previous reports, suggest that HlPrx2 protects digestive cells against membrane oxidation and suppresses unnecessary diffusion of H_2_O_2_ from midgut lumen and digestive cells. On the other hand, the midgut, ovaries, and fat bodies are known to produce vitellogenin, a phospholipoglycoprotein and a member of the lipid transfer protein superfamily that is the precursor of major yolk proteins in all oviparous organisms [26, 27]. During blood-feeding, the expression patterns of tick vitellogenin are upregulated from day 3 to engorgement; the highest expression of mRNA and protein is observed upon engorgement [27]. Vitellogenin also has a positive effect on oxidative stress resistance in bees and is a preferred target of oxidative carbonylation in comparison with hemolymph proteins in adult bees [28]. In addition, in the ovaries and fat bodies, HlPrx2 mRNA expression was upregulated from around day 3, and protein expression was present stably (Figs. 1c and 2c). This indicates HlPrx2 protein could protect vitellogenin and the organs synthesizing vitellogenin, such as the midgut, the fat bodies, and the ovaries, from the oxidative stress that occurs during blood-feeding. In the salivary glands, HlPrx2 mRNA expression was upregulated during blood-feeding (Fig. 1c), while protein expression was upregulated from unfed to partially fed states (Fig. 2c). Moreover, in the case of *HlPrx*, the other known peroxiredoxin of *H. longicornis*, mRNA is upregulated in the salivary glands, and HlPrx protein is also highly expressed in the salivary glands [12]. An anti-HlPrx antibody was detected in the host serum after several repeated tick infestations [12], suggesting that the HlPrx was released from ticks into the host eliciting to produce anti-HlPrx on immune response. In *Fasciola* parasites, infective parasites excyst from a dormant state following ingestion and penetrate the intestinal wall before migrating to the liver; in this nutrient- and oxygen-rich environment, the parasites undergo rapid growth and development, and energy is supplied by aerobic respiration [29]. This developmental situation of *Fasciola* parasites is similar to the development of ticks during blood-feeding. In addition, *Fasciola* parasites secrete Prxs into their host to regulate their environment for survival in the host body [9]. These findings strongly suggest that tick Prxs may be also secreted into the host’s body in a way similar to that of *Fasciola* parasites.

In hemocytes, *HlPrx2* mRNA expression was upregulated during blood-feeding, and a specific fluorescence was also detected in cell membranes of the hemocytes (Figs. 1c, 3). In *Ixodes ricinus*, two *Prx* homologous genes (Accession nos. AY333958 and AY333959) were strongly induced in the hemolymph after *Borrelia burgdorferi* infection [30]. Furthermore, *Borrelia* exploits the salivary Salp25D, a protein homologous to Prx in *Ixodes scapularis*, for protection against reactive oxygen intermediates generated by the mammalian neutrophils at the vector-host interface [31]. These results indicate that HlPrx2 might be related to immune response, e.g. digestion of foreign bodies such as *Borrelia* and *Babesia* parasites in hemocytes. In the mosquito *Anopheles stephensi*, 2-Cys Prx (AsPrx-4783) expression induced in the midgut was two to seven times higher in malaria parasite-infected insects than in uninfected mosquitoes [32]. Two *Prx* genes of *I. ricinus* were also induced in the midgut by *B. burgdorferi* infection [30]. HlPrx2 in the midgut may also be involved in immune response; however, further investigation is necessary.

We also performed knockdown experiments of *HlPrx* and/or *HlPrx2* genes and measured the H_2_O_2_ after the knockdown of these genes (Table 2 and Figs. 4, 5). The H_2_O_2_ concentration of no injection group in the unfed state was about 3 μM (data not shown). In comparison with insects, the H_2_O_2_ concentration in normal state silkworms was also reported at about 3 μM [33]. These observation may suggest that at a normal state, tick and silkworm H_2_O_2_ concentrations might have the same range. Therefore, this detection method of H_2_O_2_ concentration was considered as functionally acceptable. In the unfed and engorged states, the *dsDouble* group showed significantly higher concentrations of H_2_O_2_ as compared to the *dsLuc* group. These results suggest a synergistic regulation of H_2_O_2_ by HlPrx and HlPrx2. In addition, phenotype evaluation after the knockdown of *HlPrx* and/or *HlPrx2* demonstrated significant decreases in the engorged body weight, egg weight and hatching rate, particularly after *HlPrx2* knockdown. The antioxidant activities evaluated by a metal-catalyzed oxidation system seemed to be almost the same comparing 1-Cys Prx and 2-Cys Prx from the bumblebee *Bombus ignites* [34]. The donors of 1-Cys Prxs and 2-Cys Prxs are thiol and thioredoxin, respectively [7]. Thioredoxin is a major disulfide reductase system which can provide electrons to a large range of enzymes and is found to be critical for DNA synthesis and defense against oxidative stress [35]. Taken together, the 1-Cys and 2-Cys Prxs seemed to have almost the same antioxidant activity but their donors are different. These data indicate that 2-Cys Prx is more related to cell metabolism through the antioxidant activity because of its utilization of thioredoxin as donor, thus, HlPrx2 knockdown in the ticks led to the significant decrease in engorged body weight, egg weight, and hatching rate in spite of no significant effect to H_2_O_2_ concentrations in the knockdowned ticks. Therefore, these findings suggest that HlPrxs play an important role in successful blood-feeding and reproduction, with HlPrx2 being apparently more significant. Additionally, the observed effects in the *dsDouble* group were milder than those of the *dsHlPrx2* group. H_2_O_2_ can activate signaling pathways to stimulate cell proliferation, differentiation and migration in multicellular organisms [36]. These results suggest that the *dsDouble* group, but not the *dsHlPrx2* group, was exposed to a high concentration of H_2_O_2_, leading to higher engorged body weight, egg weight and hatching rate as compared to the *dsHlPrx2* group.

In endoparasites, Prx has been shown to be the most important detoxifying enzyme for their survival [8, 9] making it a candidate for use in vaccine development and a therapeutic target in treating endoparasitic infectious diseases [30, 32]. In ticks, there have been a few reports on Prxs. However, anti-HlPrx antibody was detected in the host serum after several repeated tick infestations [12], suggesting that HlPrx was released from ticks into the host and the amount of released HlPrx protein was quite small since several infestations of ticks were done to detect the anti-HlPrx antibody. In addition, ticks ingest and concentrate large amounts of the host-derived blood [23], it can be suggested that the anti-HlPrx antibody would be concentrated in tick's body. In the present study, anti-HlPrx2 antibody cross-reacted with some rabbit Prx from normal rabbit blood (see Additional file 1: Figure S1), giving some concerns whether HlPrx2 can be a good vaccine candidate. However, the knockdown of *HlPrx* and/or *HlPrx2* genes significantly affected tick blood-feeding, reproduction and antioxidant activity (Table 2 and Figs. 4, 5). Therefore, tick Prx can be a potential target for tick control and provide further understanding of the oxidative stress coping mechanisms in ticks during blood-feeding.

## Conclusion

In summary, we investigated mRNA and protein expression profiles of HlPrx2 and the localization of this protein in tick tissues. Real-time PCR showed that *HlPrx2* gene expression in whole bodies and internal organs was significantly upregulated during blood-feeding. However, protein expression was constant throughout blood-feeding. Moreover, a knockdown experiment of *HlPrx2* was performed using RNAi to evaluate its function in ticks. The knockdown of the *HlPrx2* gene caused significant differences in body weight, egg weight and hatching rate in engorged ticks as compared to those of the control group. Finally, the detection of H_2_O_2_ after the double knockdown of *HlPrxs* in ticks showed that H_2_O_2_ concentration increased before and after blood-feeding. Therefore, HlPrx2 can be considered important for successful blood-feeding and reproduction through the regulation of H_2_O_2_ concentrations in ticks during blood-feeding. This study contributes to the search for a candidate target for tick control and furthers understanding of the tick’s oxidative stress coping mechanism during blood-feeding.

## Additional files

Additional file 1:
**Figure S1.** Comparison of normal rabbit blood and engorged-state samples in developmental stages using Western blot analysis. The top arrow indicates native HlPrx2 protein, the middle arrow indicates non-specific band 1 and the bottom arrow indicates non-specific band 2 (M, marker). **Figure S2.** Confirmation of antibody’s specificity in *HlPrx* and/or *HlPrx2* genes-silencing partially fed adult ticks. Each tick’s total protein was extracted from 3 ticks pooled. The left column indicates the specific anti-serum. For loading control, tubulin was detected. (PPTX 11304 kb) Additional file 2: Candidates for non-specific bands from Japanese white rabbit blood in Western blot analysis. (DOCX 13 kb)

## Abbreviations

H_2_O_2_: Hydrogen peroxide
Prx: Peroxiredoxin
HlPrx2: *Haemaphysalis longicornis* 2-Cys peroxiredoxin
ROS: Reactive oxygen species
SDS-PAGE: Sodium dodecyl sulfate-polyacrylamide gel electrophoresis
HlPrx: *Haemaphysalis longicornis* 1-Cys peroxiredoxin
PBS: Phosphate buffered saline
IFAT: Indirect immunofluorescent antibody test
RNAi: RNA interference
*dsHlPrx2*: The double-stranded RNA of *HlPrx2*
*dsHlPrx*: The double-stranded RNA of *HlPrx*
*dsLuc*: The double-stranded RNA of the firefly *luciferase*
*dsDouble*: The double-stranded RNA mixture of *dsHlPrx* and *dsHlPrx2* at 1 μg concentration each
SA: The acinar cells of the salivary glands
SGG: The granular cells of the salivary glands
SD: The salivary duct
