# Supplementary material for: 2-Cys peroxiredoxin is required in successful blood-feeding, reproduction, and antioxidant response in the hard tick Haemaphysalis longicornis
Source: Parasit Vectors. 2016 Aug 19;9:457. doi: 10.1186/s13071-016-1748-2 (PMC4992251; doi:10.1186/s13071-016-1748-2)
Supplement: Additional file 1: — Figure S1. Comparison of normal rabbit blood and engorged-state samples in developmental stages using Western blot analysis. The top arrow indicates native HlPrx2 protein, the middle arrow indicates non-specific band 1 and the bottom arrow indicates non-specific band 2 (M, marker). Figure S2. Confirmation of antibody’s specificity in HlPrx and/or HlPrx2 genes-silencing partially fed adult ticks. Each tick’s total protein was extracted from 3 ticks pooled. The left column indicates the specific anti-serum. For loading control, tubulin was detected. (PPTX 11304 kb) [file 13071_2016_1748_MOESM1_ESM.pptx]

## Slide 1
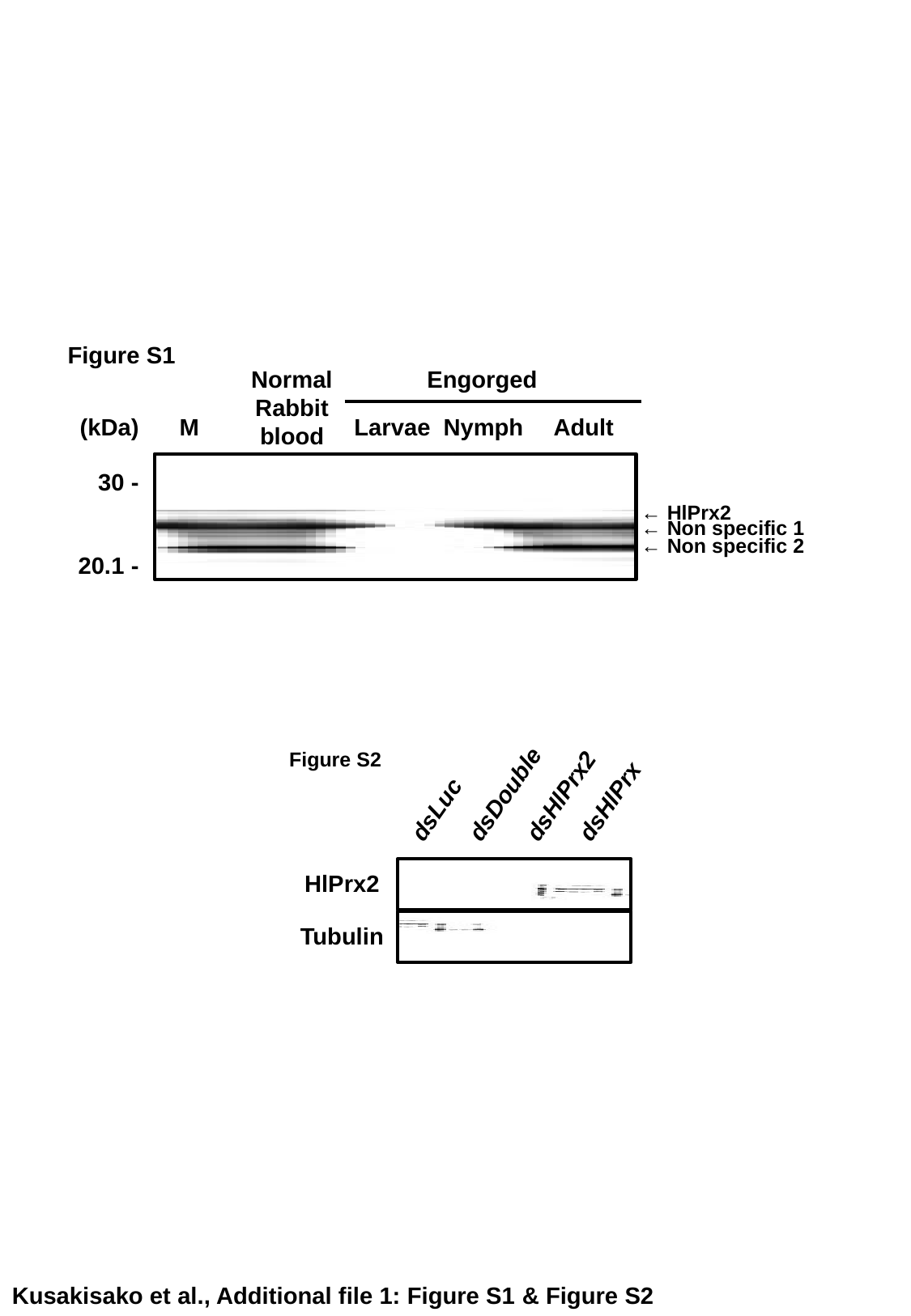

Figure S1
Normal
Rabbit
blood
Engorged
Larvae
Nymph
Adult
(kDa)
M
30 -
← HlPrx2
← Non specific 1
← Non specific 2
20.1 -
dsLuc
dsDouble
dsHlPrx2
dsHlPrx
HlPrx2
Tubulin
Figure S2
Kusakisako et al., Additional file 1: Figure S1 & Figure S2
