# Supplementary material for: 2-Cys peroxiredoxin is required in successful blood-feeding, reproduction, and antioxidant response in the hard tick Haemaphysalis longicornis
Source: Parasit Vectors. 2016 Aug 19;9:457. doi: 10.1186/s13071-016-1748-2 (PMC4992251; doi:10.1186/s13071-016-1748-2)
Supplement: Additional file 2: — Candidates for non-specific bands from Japanese white rabbit blood in Western blot analysis. (DOCX 13 kb) [file 13071_2016_1748_MOESM2_ESM.docx]

Candidates for non-specific bands from Japanese white rabbit blood in western blot analysis

| Candidate protein* | Predicted  molecular weight  (kDa)** | Isoelectric  point** | Calculated  molecular weight  (kDa)*** | Identity  with  HlPrx2* | Accession no.* |
| --- | --- | --- | --- | --- | --- |
| Peroxiredoxin 1 | 22 | 8.2 | 23  (Non-specific 1) | 77% | XP_002715184 |
| Thioredoxin-dependent  peroxide reductase | 28 | 8.3 | 24  (Non-specific 2) | 67% | XP_002718732 |
| HlPrx2 | 22 | 6.8 | 26 | - | LC049075 |

* The deduced amino acid translation of the *HlPrx2* gene sequence was determined using GENETYX version 7.0 software (GENETYX, Tokyo, Japan). A BLAST server (http://blast.ncbi.nlm.nih.gov/Blast.cgi) was used to search homologous genes from GenBank (http://www.ncbi.nlm.nih.gov/genbank).

** The theoretical molecular mass and isoelectric points were calculated using a ProtParam tool (http://web.expasy.org/protparam/).

*** The calculated molecular weight was assessed using the FluorChem^®^FC2 software (Alpha Innotech Hessisch Oldendorf, Germany) band analysis tool.

**Kusakisako et al., Additional file 2**
